# Lyophilized Amniotic Membrane Eye Drops Stabilize the Tear Film in Dry Eye Disease: A Prospective Cohort Study

**DOI:** 10.3390/jcm15103920

**Published:** 2026-05-19

**Authors:** Jelena Kostic, Svetlana Stanojlovic, Natasa Maksimovic, Vladimir Milutinovic, Nada Avram, Tanja Kalezic, Bojana Dacic Krnjaja, Borivoje Savic

**Affiliations:** 1 Clinic for Eye Diseases, University Clinical Center of Serbia, 11000 Belgrade, Serbia; 2 Faculty of Medicine, University of Belgrade, 11000 Belgrade, Serbia; 3 Institute of Epidemiology, Faculty of Medicine, University of Belgrade, 11000 Belgrade, Serbia; 4 Faculty of Medicine Foča, University of East Sarajevo, 73300 Foča, Bosnia and Herzegovina

**Keywords:** dry eye disease, amniotic membrane, tear break-up time, HC-HA/PTX3, extracellular matrix

## Abstract

**Purpose**: To evaluate the clinical efficacy and safety of eye drops containing lyophilized amniotic membrane (AM) in the treatment of dry eye disease (DED), with a focus on tear film stabilization and epithelial–immune balance. Methods: In this prospective cohort study, 40 patients (80 eyes) with DED were followed over six visits. The primary outcome was tear break-up time (TBUT). Secondary outcomes included corneal and conjunctival staining graded by the Oxford scale, meibomian gland parameters, corneal sensitivity (Cochet–Bonnet esthesiometry), best-corrected visual acuity, intraocular pressure (IOP), and Schirmer I test. Continuous variables were analyzed using repeated-measures ANOVA with Greenhouse–Geisser correction and Bonferroni post hoc testing; ordinal outcomes were analyzed using the Friedman test with Dunn–Bonferroni correction. **Results**: TBUT increased significantly in both eyes (OD: +5.3 s; OS: +4.9 s; both *p* < 0.001; ηp^2^ ≈ 0.33). Corneal and conjunctival staining scores decreased (*p* < 0.001), meibomian gland quality and expressibility improved (*p* < 0.001), and corneal sensitivity increased (*p* < 0.001), while visual acuity and IOP remained stable. Schirmer I values showed no significant change. The combined pattern of changes (TBUT ↑, staining ↓, meibum/expressibility ↑, sensitivity ↑) indicates tear film stabilization and ocular surface improvement with a preserved safety profile. **Conclusions**: Lyophilized AM eye drops significantly prolong TBUT and improve clinical signs of DED, presumably by restoring the extracellular matrix (ECM) niche and the heavy-chain hyaluronan/pentraxin 3 (HC-HA/PTX3) complex, reducing proteolytic burden, and promoting a pro-resolving immune balance, with potential neurotrophic effects. These findings support the adjunctive use of AM-derived eye drops within contemporary TFOS DEWS II-based management algorithms for dry eye disease.

## 1. Introduction

Amniotic membrane-based eye drops represent a novel regenerative approach to the treatment of dry eye disease, aimed at restoring ocular surface homeostasis through extracellular matrix-mediated epithelial support rather than through classical anti-inflammatory or tear-substituting mechanisms. The rationale for their use in our study is based on the assumption that preserved extracellular matrix components, growth factors, and immunomodulatory mediators can improve epithelial integrity, neurosensory function, and tear film stability, providing a biologically grounded therapeutic strategy for dry eye disease. Dry eye disease (DED) is a disorder of tear film homeostasis with a heterogeneous etiology and a shared outcome of tear film destabilization, disruption of the epithelial barrier, and variable inflammatory activation. The TFOS DEWS II reports emphasize the interplay between evaporative loss and hyperosmolarity, epithelial activation, innate and adaptive immune responses, and neurosensory dysfunction, collectively leading to reduced tear break-up time (TBUT) and ocular surface damage [1,2]. Tear fluid in patients with DED frequently contains elevated levels of proinflammatory cytokines (IL-6, IL-1β, TNF-α) and increased proteolytic activity, correlating with disease severity and subjective symptoms, whereas IL-10 is associated with a pro-resolving/tolerogenic response. The biomarker profile depends on the disease phenotype and stage [3,4,5,6,7,8,9,10]. The contemporary concept of DED highlights the importance of modulating the epithelial–immune balance as a therapeutic target. The amniotic membrane exhibits a unique extracellular matrix architecture enriched in heavy-chain hyaluronan/pentraxin-3 complexes, collagens, and laminins, which collectively create a pro-regenerative niche, limit fibrotic remodeling, and reduce proteolytic burden [11,12,13,14,15,16]. Experimental and clinical evidence demonstrates that amniotic membrane and its derivatives modulate epithelial activation and NF-κB/MAPK signaling pathways, attenuate IL-6-driven inflammation, and shift the local microenvironment toward a pro-resolving profile, including IL-10-mediated effects, thereby supporting re-epithelialization and tear film stability [11,12,13,14,15,16]. In parallel, growth and neurotrophic factors such as EGF, HGF, KGF, and NGF may contribute to the restoration of neurosensory control of blinking and subjective ocular comfort, which is reflected clinically by improved TBUT and reduced symptoms [17]. On this biological basis, eye drops containing lyophilized amniotic membranes are conceptually designed to target tear film quality and epithelial barrier integrity rather than primarily increasing the aqueous volume [1,2,18,19,20,21,22,23,24]. In our study, we evaluated the clinical efficacy and safety of lyophilized amniotic membrane eye drops in patients with DED, with TBUT as the primary clinical endpoint and a composite of ocular surface stabilization parameters (fluorescein and vital staining, meibomian gland function, corneal sensitivity) as key secondary outcomes. We hypothesized that this therapeutic approach would preferentially improve tear film quality and epithelial barrier function, with a limited impact on aqueous tear production [1,2,18,19,20,21,22,23,24]. Based on existing literature, we additionally considered the epithelial–immune balance (IL-6/IL-10) and proteolytic burden, including matrix metalloproteinase activity, as plausible biological underpinnings of the observed clinical effects [3,4,5,6,7,8,9,10,25,26].

## 2. Materials and Methods

### 2.1. Study Design and Participants

This was a prospective cohort study conducted at the Clinic for Eye Diseases, University Clinical Center of Serbia (UCCS), from October 2024 to July 2025. A total of 40 patients (80 eyes) aged ≥18 years with a confirmed diagnosis of dry eye disease (DED) were enrolled. Sample size calculation was performed at the patient level using G*Power software (version 3.1.9.7; Heinrich Heine University Düsseldorf, Düsseldorf, Germany); assuming an effect size f = 0.33, α = 0.05, and power = 0.95, the minimum required size was 33 patients. To allow for 20% attrition, 40 patients were included.

### 2.2. Inclusion and Exclusion Criteria Inclusion Criteria

Following TFOS DEWS II diagnostic criteria, patients were included if they had an OSDI score ≥ 13 and at least one objective sign of homeostatic disruption (TBUT < 10 s or corneal staining > 5 spots). Additional inclusion criteria were: age ≥ 18 years; use of topical cyclosporine A (CsA) for the preceding 3 months; daily computer use ≥1 h; and signed informed consent. Exclusion criteria: ocular surgery within 6 months; known hypersensitivity to topical preparations; use of topical corticosteroids within the previous month; active ocular infection; pregnancy/lactation; immunodeficiency; or contact lens wear.

### 2.3. Ophthalmic Examinations

Clinical assessments were performed at baseline and after each 14-day treatment cycle for a total of six visits. Patients were instructed not to instill any eye drops for at least 6 h before each visit. Tear break-up time (TBUT) was measured at each visit at least 2 h after the most recent instillation of any lubricant to minimize immediate artificial stabilization effects.

The examination protocol included:

1. Best-corrected visual acuity (Snellen chart).
2. TBUT assessment (cutoff 10 s).
3. Corneal fluorescein staining graded using the Oxford scale (0–4) [27].
4. Conjunctival staining with vital dyes graded using the Oxford scale [27].
5. Evaluation of meibomian gland expressibility and meibum quality (0–3).
6. Schirmer I test without anesthesia.
7. Slit-lamp examination of the anterior and posterior segments.
8. Corneal sensitivity measurement (Cochet–Bonnet esthesiometer).
9. Intraocular pressure (IOP) via Goldmann applanation tonometry.

### 2.4. Preparation of Lyophilized Amniotic Membrane Eye Drops

Amniotic membranes (AMs) were obtained following elective cesarean sections at the Gynecology and Obstetrics Clinic “Narodni front” after donor consent and serological screening (HBV, HCV, HIV). Tissue processing was conducted at the licensed UCCS Eye Bank. AM tissue was immersed in antibiotics/antimycotics for 24 h, rinsed, and the amnion was separated from the chorion. Fragments were lyophilized using a Lyovac GT 2 device (Institute for Biological Research “Siniša Stanković”, Belgrade, Serbia). The obtained powder was reconstituted (5 g powder per 10 mL sterile water for injection) and filtered through a 0.22 μm membrane (Versapor, PALL Medical, New York, NY, USA).

### 2.5. Product Quality Control and Stability

Preparation was performed in a Class II laminar flow hood. Pre-release checks included visual clarity inspection (no visible particles) and pH measurement (target range 6.5–7.8). Real-time stability monitoring on days 0, 7, and 14 ensured the assigned 14-day shelf-life. Formulated solutions were stored at +5 °C and protected from light. A dedicated batch log ensured full traceability of all materials and operators.

### 2.6. Intervention and Statistical Analysis

Patients received AM drops 6 times daily (2 drops per eye) for 14 days per cycle. Concomitant therapy (lubricants and CsA) remained constant throughout the study. Statistical analysis was performed at the eye level. Normality was assessed using the Shapiro–Wilk test. For the primary outcome (TBUT), repeated-measures ANOVA (RM-ANOVA) with Greenhouse–Geisser correction and Bonferroni post hoc testing was applied. Ordinal outcomes (Oxford scores, meibomian parameters) were analyzed using the Friedman test with Dunn–Bonferroni correction. Continuous secondary variables were analyzed using RM-ANOVA with partial η^2^ as effect size. To address potential Type I error from the non-independence of data from both eyes, a sensitivity analysis was performed using only the right eye (OD) of each participant (*N* = 40). A two-sided *p* value < 0.05 was considered statistically significant.

## 3. Results

A total of 80 eyes from 40 patients were analyzed over six visits (baseline plus five follow-up visits). Tear break-up time (TBUT) increased significantly over time in both eyes (RM-ANOVA with Greenhouse–Geisser correction: F(3.9, 304) = 38.7, *p* < 0.001; ηp^2^ = 0.33). Post hoc comparisons (Bonferroni-adjusted) demonstrated significant improvements versus baseline from the third visit onwards (all *p* < 0.001; Table 1). Mean (±SD) TBUT increased from 6.8 ± 2.0 s to 12.1 ± 2.0 s in the right eye and from 7.0 ± 2.0 s to 11.9 ± 2.0 s in the left eye. To address the potential risk of Type I error arising from the non-independence of data from both eyes, a sensitivity analysis using only the right eye (OD) of each participant (*N* = 40) was performed, and the results for the primary outcome (TBUT) remained statistically significant (*p* < 0.001).

A summary of secondary outcomes is presented in Table 2. Corneal and conjunctival staining scores decreased significantly between the baseline and the final visit (cornea: 2 [1–3] to 1 [1–2]; conjunctiva: 2 [1–2] to 1 [0–2]; both *p* < 0.001; W = 0.32 and 0.28, respectively). Meibomian gland parameters also showed significant changes over the treatment period, with improvements in meibum quality (1 [1–2] to 2 [2–3]; *p* < 0.001; W = 0.30) and expressibility (1 [1–2] to 2 [1–3]; *p* < 0.001; W = 0.26).

Corneal sensitivity (Cochet–Bonnet esthesiometry) increased from 45.1 ± 7.8 mm at baseline to 55.3 ± 8.2 mm at the final visit (*p* < 0.001; ηp^2^ = 0.11). Best-corrected visual acuity and intraocular pressure (IOP) remained stable throughout the study period (*p* = 0.31 and *p* = 0.66; ηp^2^ = 0.02 and 0.01, respectively). Schirmer I values changed from 12.3 ± 4.6 mm at baseline to 13.1 ± 4.7 mm at the final visit, and this change was not statistically significant (*p* = 0.08; ηp^2^ = 0.03).

## 4. Discussion

In this prospective cohort of 40 patients (80 eyes), we observed a robust and clinically meaningful improvement in TBUT of approximately 5 s (*p* < 0.001; partial ηp^2^ ≈ 0.33), accompanied by stable safety parameters (visual acuity, IOP, Schirmer I). The consistent prolongation of TBUT in both eyes indicates a clinically meaningful stabilization of the tear film primarily through improvement of ocular surface quality—specifically the epithelial barrier, glycocalyx, and mucin–lipid interface—rather than an increase in aqueous tear volume. This pattern suggests that lyophilized amniotic membrane (AM) eye drops primarily act by improving tear film quality and stability, potentially through the restoration of tight junction integrity, rather than by substantially increasing aqueous volume [1,2,17,18]. These findings align with the TFOS DEWS II diagnostic and therapeutic framework, which recognizes TBUT and ocular surface signs as valid treatment targets and outcome measures [18,19,20].

The hypothesized biological underpinnings of our results are supported by the established literature regarding the extracellular matrix (ECM) architecture of AMs. It is plausible to suggest that the hyaluronan-rich matrix and heavy-chain hyaluronan/pentraxin 3 (HC-HA/PTX3) complexes, together with collagens and laminins, may provide a pro-regenerative niche that mechanically and biochemically protects the epithelium, reduces proteolytic stress (including matrix metalloproteinase-mediated degradation), and promotes organized re-epithelialization [3,4,5,6,24]. In parallel, the presence of growth and neurotrophic factors (EGF, HGF, KGF, NGF) is well-documented in AM-derived products and likely contributes to epithelial cell migration and neurotrophic stabilization of the cornea, which would be consistent with the observed increase in corneal sensitivity and improved comfort in our patients [6,19].

From an immunological perspective, based on the known properties of AMs, these preparations are thought to favor the resolution of inflammation and restoration of homeostasis rather than simple immunosuppression. While DED is frequently associated with elevated tear levels of IL-6, IL-1β, and TNF-α [8,9,10,11], existing literature suggests that AM derivatives, through HC-HA/PTX3 and ECM-mediated signaling, may attenuate epithelial activation and promote a pro-resolving immune balance, potentially characterized by a shift in the IL-6/IL-10 ratio [14,21,22,23]. Although our study did not directly measure these biomarkers, such mechanisms offer a coherent and literature-based explanation for the improved epithelial integrity and prolonged TBUT observed in this cohort.

Our findings are directionally consistent with existing clinical and real-world data on AM extracts and AM-derived eye drops (AME/AMED), which report improvements across heterogeneous formulations [6,13,14,15]. Parallel reductions in Oxford staining scores and improvements in meibomian gland quality and expressibility support the concept of reduced proteolytic burden and a more functional lipid layer. At a conceptual level, the restoration of the ECM niche and epithelial barrier emerges as a central mode of action. Based on established biological models, shifts in cytokine balance and reduced proteolytic burden provide a plausible mechanistic framework for the tear film stabilization we recorded [8,9,10,11]. It is also conceivable that interindividual differences in these pathways (e.g., MMPs, collagen-related signaling) may modulate responsiveness to AM-based therapies [1,2,16].

Within the TFOS DEWS II treatment algorithm, AM eye drops integrate naturally into multimodal management strategies. A critical consideration in our study is the concomitant use of lubricants and topical Cyclosporine A (CsA). While the ongoing use of these therapies could complicate the isolation of the AM effect, it is important to note that all enrolled patients had demonstrated an insufficient clinical response to both CsA and lubricants for at least 3 months prior to the initiation of AM drops. Since the dosage of CsA remained constant for all patients throughout the study period, the observed significant improvement following the addition of lyophilized AM eye drops suggests a clear adjunctive benefit, providing clinical value beyond what was achieved with standard therapy alone [18,19,20].

Overall, our results are consistent with the hypothesized effects of AM/AME—ECM-mediated regeneration, pro-resolving immunomodulation, and potential neurotrophic support—thus offering a biologically grounded rationale, based on known properties of AMs, for the adjunctive use of lyophilized AM eye drops in contemporary DED management [1,2,23,24,25,26].

In addition to aligning with the theoretical biological framework of AM-based therapies, our study provides several clinical insights. A mean TBUT improvement of approximately 5 s represents a significant effect size. The increase in corneal sensitivity is compatible with potential neurotrophic support provided by AM-derived factors. Furthermore, the dissociation between TBUT recovery and Schirmer I scores reinforces the concept that AM may be particularly effective in phenotype-specific subgroups of DED where epithelial instability dominates over lacrimal gland insufficiency.

### Limitations

**We Acknowledge Several Limitations to This Study.** The lack of a control group and the continued use of Cyclosporine A make it challenging to isolate the effect of AM eye drops in a vacuum; however, the “insufficient responder” status of our cohort prior to enrollment mitigates this to an extent. Furthermore, analyzing 80 eyes from 40 patients as independent samples may increase the risk of Type I error. To address this, we conducted a sensitivity analysis using only one eye per patient, which yielded consistent and statistically significant results, supporting the validity of our conclusions. Finally, the lack of direct biomarker quantification means that the discussed molecular pathways (HC-HA/PTX3, IL-6/IL-10 balance, and MMP reduction) remain hypothesized biological underpinnings based on established literature rather than direct findings of this study. Future research incorporating proteomic analysis and a randomized controlled design is needed to confirm these specific biological shifts and further isolate the therapeutic impact of AMs. Additionally, the single-arm design does not account for a potential placebo effect, and since the study was conducted in a specialized tertiary center, the generalizability of these results to broader or milder DED populations may be limited.

## 5. Conclusions

In this prospective cohort, eye drops containing lyophilized amniotic membrane were associated with consistent and clinically relevant prolongation of TBUT, improvement in ocular surface staining and meibomian gland function, and increased corneal sensitivity, while best-corrected visual acuity, IOP, and Schirmer I values remained stable. The overall response pattern indicates that the primary contribution of this therapy is stabilization of tear film quality—via restoration of epithelial barrier function and a more competent mucin–lipid layer—rather than augmentation of aqueous tear production. Such an epithelial- and ECM-centered mode of action, together with a likely pro-resolving immunomodulatory profile, supports the use of lyophilized AM eye drops as a rational adjunct within TFOS DEWS II-based treatment algorithms, particularly in patients with evaporative DED and meibomian gland dysfunction insufficiently controlled by standard therapy. Taken together, lyophilized AM eye drops represent a biologically grounded, clinically promising adjunctive option for improving tear film stability and ocular surface outcomes in carefully selected patients with dry eye disease.

## Figures and Tables

**Table 1:** Change in TBUT between Baseline and Final Visit.

Parameter	Baseline (Mean ± SD)	Final Visit (Mean ± SD)	Δ (s)	Test/*p*-Value
TBUT OD (s)	6.8 ± 2.0	12.1 ± 2.0	5.3	RM-ANOVA (GG)/<0.001
TBUT OS (s)	7.0 ± 2.0	11.9 ± 2.0	4.9	RM-ANOVA (GG)/<0.001

Note: TBUT–tear break-up time; OD–right eye; OS–left eye; SD–standard deviation; RM-ANOVA (GG)–repeated-measures ANOVA with Greenhouse–Geisser correction and Bonferroni-adjusted post hoc testing. Effect size for the overall TBUT change across visits: partial η^2^ = 0.33.

**Table 2:** Summary of Secondary Outcomes (*N* = 80 eyes).

Outcome	Baseline	Final Visit	Test/*p*-Value	Effect Size
Oxford cornea (0–4)	2 [1–3]	1 [0–2]	Friedman/<0.001	W = 0.32
Oxford conjunctiva (0–4)	2 [1–2]	1 [0–2]	Friedman/<0.001	W = 0.28
Meibum quality (0–3)	1 [1–2]	2 [2–3]	Friedman/<0.001	W = 0.30
Expressibility (0–3)	1 [0–2]	2 [1–3]	Friedman/<0.001	W = 0.26
Cochet–Bonnet (mm)	45.1 ± 7.8	55.3 ± 8.2	RM-ANOVA/<0.001	ηp^2^ = 0.11
Visual acuity (logMAR)	0.20 ± 0.12	0.15 ± 0.11	RM-ANOVA/0.31	ηp^2^ = 0.02
Schirmer I (mm/5 min)	12.3 ± 4.6	13.1 ± 4.7	RM-ANOVA/0.08	ηp^2^ = 0.03
IOP (mmHg)	14.5 ± 2.6	14.3 ± 2.5	RM-ANOVA/0.66	ηp^2^ = 0.01

Notes: Values for Oxford scores and meibomian parameters are presented as median [interquartile range]. IOP–intraocular pressure; RM-ANOVA–repeated-measures ANOVA; W–Kendall’s W; ηp^2^–partial eta squared.

## Data Availability

The original contributions presented in this study are included in the article. Further inquiries can be directed to the corresponding author(s).
